# Surgical Outcomes after Radiotherapy in Rectal Cancer

**DOI:** 10.3390/cancers16081539

**Published:** 2024-04-18

**Authors:** Sofieke J. D. Temmink, Koen C. M. J. Peeters, Per J. Nilsson, Anna Martling, Cornelis J. H. van de Velde

**Affiliations:** 1 Department of Molecular Medicine and Surgery, Karolinska Institutet, 171 76 Stockholm, Sweden; 2 Department of Surgery, Leiden University Medical Center, 2333 ZA Leiden, The Netherlands

**Keywords:** rectal cancer, radiotherapy, neoadjuvant treatment, surgical outcomes

## Abstract

**Simple Summary:**

In recent years, there have been significant improvements in the treatment of rectal cancer. Preoperative treatment and surgical techniques have advanced, leading to fewer local recurrences, fewer surgical complications, and more complete resections. Recent trials investigating total neoadjuvant treatment aim to decrease distant metastasis and improve overall survival in patients with locally advanced rectal cancer. In addition, adding chemotherapy to the preoperative treatment increases the complete response rate to 30 to 50 percent of patients. This means that no residual tumour is left and that patients can follow a Watch & Wait surveillance program, potentially avoiding surgery, complications, and colostomy. Nonetheless, many factors have to be considered when deciding on a treatment plan for patients with rectal cancer. Ongoing research aims to identify the optimal treatment regimen, considering factors like tumour stage, toxicity of (neo)adjuvant treatment, and patient preference.

**Abstract:**

Over the past decade, the treatment of rectal cancer has changed considerably. The implementation of TME surgery has, in addition to decreasing the number of local recurrences, improved surgical morbidity and mortality. At the same time, the optimisation of radiotherapy in the preoperative setting has improved oncological outcomes even further, although higher perineal infection rates have been reported. Radiotherapy regimens have evolved through the adjustment of radiotherapy techniques and fields, increased waiting intervals, and, for more advanced tumours, adding chemotherapy. Concurrently, imaging techniques have significantly improved staging accuracy, facilitating more precise selection of advanced tumours. Although chemoradiotherapy does lead to the downsizing and -staging of these tumours, a very clear effect on sphincter-preserving surgery and the negative resection margin has not been proven. Aiming to decrease distant metastasis and improve overall survival for locally advanced rectal cancer, systemic chemotherapy can be added to radiotherapy, known as total neoadjuvant treatment (TNT). High complete response rates, both pathological (pCR) and clinical (cCR), are reported after TNT. Patients who follow a Watch & Wait program after a cCR can potentially avoid surgical morbidity and colostomy. For both early and more advanced tumours, trials are now investigating optimal regimens in an attempt to offer organ preservation as much as possible. Multidisciplinary deliberation should include patient preference, treatment toxicity, and likelihood of end colostomy, but also the burden of intensive surveillance in a W&W program.

## 1. Introduction

In the past few decades, the management of rectal cancer has changed substantially. During this time, research has focused on the optimal treatment regimens for patients with rectal cancer. In the early days, local control was a big challenge with high morbidity and mortality rates. The implementation of total mesorectal excision (TME) surgery has improved survival rates and reduced local recurrence rates significantly and enables surgeons to achieve sphincter preservation more often [1,2]. TME surgery remains the standard of care worldwide [3]. However, treatment strategies, mainly concerning (neo)adjuvant radiotherapy and chemotherapy, are still rapidly evolving, and organ preserving strategies are gaining ground. 

Preoperative radiotherapy has proven its value in reducing local recurrences [4], and even in terms of overall survival (OS) rates before TME was the standard of care [5]. Since then, trials investigating neoadjuvant treatment have evolved, aiming to decrease distant metastasis and eventually OS. Adding systemic chemotherapy to the preoperative treatment, known as total neoadjuvant treatment (TNT), is increasingly being used [6].

Another important aim of current rectal cancer trials is to find optimal treatment algorithms for achieving organ preservation. A well-established advantage of TNT encapsulates the high percentages of pathological complete response (pCR) rates [7,8]. When already diagnosed in the preoperative setting, this can spare patients surgery altogether, avoiding surgical morbidity and, in some cases, colostomy. 

However, neoadjuvant treatment does not come for free, and the positive impact on the oncological outcome should be carefully balanced against the toxicity of the treatment, surgical outcomes, and QoL. All these factors have to be taken into account when defining the optimal treatment plan for the individual patient with rectal cancer.

The aim of this review is to highlight important trials investigating the role of radiotherapy as part of a multimodality treatment in rectal cancer patients, focusing on surgical implications, complications, and outcomes.

## 2. Surgical Approach 

Negative resection margins are important predictors for local recurrence in rectal cancer [9,10]. Before TME became the standard of care, conventional surgery meant the blunt dissection of the rectal fascia, resulting in the incomplete removal of mesorectal tissue. The importance of a clear margin became increasingly recognized, showing that the incomplete excision of the tumour at the circumferential resection led to much higher risks of local recurrence [9,11]. During TME surgery, first described by Bill Heald, the mesorectal fat is dissected, including all its lymph nodes [1]. This technique results in high rates of negative resection margins and has decreased the number of local recurrences considerably [1,2,4]. 

Another advantage observed after the implementation of TME surgery is that it significantly decreased the number of abdominoperineal resections (APRs) and concomitant permanent colostomy rates [2]. APR surgery is often required for distal tumours, but with the TME technique, dissection under direct vision down to the pelvic floor is feasible, facilitating an (ultra)low anastomosis. An end-colostomy and low pelvic anastomosis are both potential outcomes after surgical resection that can drastically impact and impair a patient’s Quality of Life (QoL) [12]. 

Postoperative morbidity and mortality rates have decreased after the implementation of TME surgery, but are still substantial. The most severe complication reported after surgery is anastomotic leakage, which can lead to peritonitis, emergency relaparotomy, and increased mortality rates. Additionally, patients can suffer from symptoms due to impaired anorectal function after sphincter-preserving surgery, known as low anterior resection syndrome (LARS). Other complications that are often reported after TME surgery are wound healing disorders and infections. In addition to a reduction in the QoL, it is acknowledged that surgical complications are associated with reduced local recurrence-free survival (LRFS) and distant metastasis-free survival (DMFS) and OS [13].

## 3. Pre- and Postoperative Radiotherapy

### 3.1. Preoperative Radiotherapy

In the early days, local recurrence for rectal cancer surgery was common, with rates of up to 30% [14,15]. The implementation of radiotherapy, with the first trials starting in 1980, has significantly reduced this rate. Radiation schedules with conventional fractionation (1.8–2.0 Gy) and short-term treatment with 5 Gy as the fraction size have been extensively studied [16]. The European Organization for Research and Treatment of Cancer (EORTC) trial and Stockholm I trial were among the first large trials to show a positive effect of radiotherapy followed by surgery vs. surgery alone regarding the local control. Both reported a significant decrease after radiotherapy in a neoadjuvant setting while no differences in DM or OS were found [14,17]. The EORTC trial (34.5 Gy in 19 days overall) reported a 5 year LRFS of 85% vs. 65% (*p* < 0.001). The Stockholm I trial (25 Gy in 5 days) found a similar difference in local recurrence of 15% vs. 30% (HR 0.51, *p* < 0.01) [17]. However, after radiotherapy, the Stockholm I trial found higher postoperative morbidity, mainly because of wound infections and wound dehiscence, and also a concernedly higher mortality rate (2% vs. 8%, *p* < 0.01). Important to note is that this difference was restricted to patients older than 75 years and was mainly due to cardiovascular deaths, which was also seen in a small trial from St. Mark’s Hospital [18] while other trials at this time did not report these differences [14,19].

Aiming to lower the postoperative mortality rate after preoperative radiotherapy, the Swedish Rectal Cancer trial, partly overlapping with the Stockholm II trial, used the same radiotherapy schedule as in Stockholm I, but with three or four radiation portals (compared to only two beams in the Stockholm I trial) that had a relatively smaller target field, radiating less tissue outside the tumour [5,20]. Additionally, an upper age limit of 80 was introduced. A significant difference was reported in local recurrence rates (11% vs. 26%, *p* < 0.001) and there was no difference in postoperative mortality anymore. The results of the TME trial (see below) demonstrated that this same radiotherapy schedule was safe even in patients older than 80 years undergoing TME surgery [4].

As mentioned above, the oncological benefit of a preoperative radiotherapy regimen of 25 Gy in 5 days was proven in multiple trials, and this regimen is still used today, mainly in Europe. In 2001, the Dutch TME trial confirmed this benefit also after the standardisation of the TME technique, with a 2-year local recurrence rate of 2.4% vs. 8.2% in the surgery-alone group (*p* < 0.001) [21]. Long-term results from the Swedish Rectal Cancer trial now showed a clear survival benefit while, also after 12 years, the TME trial could not demonstrate any survival differences between the two arms [4,5]. Although radiotherapy significantly improved cancer-specific survival in patients with a negative resection margin, an increase in the occurrence of other causes of death, mainly secondary malignancies, after radiotherapy resulted in an equal OS rate [4]. Additionally, the lower absolute difference in recurrence rate after TME surgery might result in not having enough events to significantly impact OS rates. 

One of the most important surgical outcomes with a known prediction in terms of oncological outcomes is the circumferential margin [9,11]. In the TME trial, the positive effect of radiotherapy on the local control was primarily seen in patients with a negative resection margin [4]. This could explain why, in subgroup analysis, radiotherapy did not seem to have an effect on distal tumours, with positive resection margins being as high as 30% in the APR group compared to 11% in the LAR group (*p* < 0.001). 

### 3.2. Postoperative Radiotherapy

The delivery of postoperative radiotherapy compared with surgery alone has yielded more doubtful results. A Danish trial [22] and GITSG trial [23] compared the delivery of 40–50 Gy in adjuvant settings to surgery alone and found no benefit in terms of the local control or survival, or only a benefit in certain stages of rectal cancer. A few years later, the MRC III trial did report a positive influence of adjuvant radiotherapy on the local control compared to surgery alone (HR 0.54 [0.38–0.77], *p* = 0.001) [24]. No trial has reported a survival benefit. The latter did not find a difference in the occurrence of late bowel complications or overall complication rate. 

### 3.3. Preoperative vs. Postoperative Radiotherapy

The Uppsala trial was the first to randomise patients between pre- and postoperative radiotherapy regimens [13]. Even with a higher dose in the postoperative setting (60 Gy in a total of 8 weeks), it is interesting that a statistically significantly lower local recurrence rate in favour of the preoperative radiotherapy was found (*p* = 0.02) [25]. An important reason for this difference was the higher compliance rate in the preoperative group, since 50% of the patients in the postoperative treatment group were reported not to commence treatment until more than 6 weeks after surgery, mainly because of delayed wound healing and/or postoperative fatigue.

Another presumed reason, in addition to compliance, for the better oncological results after preoperative radiotherapy is this therapy’s stronger effect in a surgically non-disturbed tumour bed: without surgery, tumour cells in the periphery are better oxygenated, and thus better targeted, than after surgery. Moreover, magnetic resonance imaging (MRI) significantly improved imaging, which led to more accurate staging and selection of high-risk tumours in a preoperative setting, making neoadjuvant therapy more appealing. 

Most large trials investigating preoperative radiotherapy (EORTC, Stockholm I, TME) found an increased frequency of infections and/or dehiscence in the perineal wounds after abdominoperineal resection, with a prolonged hospital stay as a consequence [13,14,26] (Table 1). With the perineum being in the radiation field, the Uppsala trial reported rates of perineal wound sepsis of 33% in the preoperative radiotherapy group vs. 18% in the postoperative group (*p* < 0.01) [25]. No trial has reported an increase in the number of anastomotic problems after preoperative radiotherapy when compared to postoperative radiotherapy or surgery alone [26,27]. 

The Uppsala trial reported an increased risk of late small bowel obstruction after postoperative irradiation when compared to preoperative irradiation (11% vs. 5%) and surgery alone (6%) (*p* < 0.01) [25] (Table 1). This trend has also been reported in other trials comparing postoperative radiotherapy with surgery alone [22,23]. A combined analysis of all patients from the Stockholm I and II trials also found a significant difference in intestinal obstruction in preoperative irradiated patients (13.3% vs. 8.5%), mainly after 2 years of follow-up [28]. Interestingly, this difference was only seen for patients from the Stockholm I trial, indicating that this late effect can mainly be explained by the larger volume used, with more of the small bowel in the radiation field. No difference in bowel obstruction was found in the TME trial [29]. 

**Table 1:** Reported complications after surgery from main clinical studies using 5 × 5 Gy as preoperative radiotherapy treatment.

	No	Randomisation	Overall Complications	All Wound Infections	Perineal Wound Complications APR	Anastomotic Leakage or Dehiscence	Bowel Obstruction	Postoperative Mortality
Preoperative radiotherapy vs. surgery								
Stockholm I (1980–1987) [15,28]	424	RT + surgery	28% *	14% ^#^		2%	11%	8% *
	425	surgery	20%	9%		3%	7%	2%
Swedish RCT (1987–1990) [30]	553	RT + surgery	44% *		20% *	11%		4%
	557	surgery	34%		10%	8%		3%
Stockholm II (1987–1993) [20,28]	272	RT + surgery	41% *	15% ^#$^		4%	9%	2%
	285	surgery	28%	6%		3%	8%	1%
TME trial (1996–1999) [31]	924	RT + surgery	48% *		29% *	11%	11%	4%
	937	surgery	41%		18%	12%	11%	3%
Preoperative vs. postoperative radiotherapy								
Uppsala trial (1980–1985) [13]	236	RT + surgery			33% *	14%	5% *	3%
	235	surgery + RT			18%	13%	11%	5%
Immediate surgery vs. delay								
Stockholm III (1998–2013) [32]	357	RT + direct surgery	53%*	22%		6%	11%	<1%
	355	RT + delayed surgery	41%	17%		6%	9%	<1%

* *p*-value < 0.05; ^#^ unknown *p*-value; ^$^ majority of infections occurred in the perineal wound after abdominoperineal resection. RT = radiotherapy.

## 4. Chemoradiation

In the postoperative setting, results after radiotherapy combined with chemotherapy were more convincing than those of postoperative radiotherapy alone. The GITSG trial, a four-arm study with a surgery-alone arm, postoperative radiotherapy, postoperative chemotherapy, and postoperative combined radiotherapy and chemotherapy arm, showed superior survival and local recurrence rates in the combined modality arm vs. the surgery-only arm [23]. These and other results led postoperative radiotherapy combined with chemotherapy to become the standard of care in the US after a National Institute of Health consensus conference in 1990 [33]. 

As mentioned, staging became much more precise when MRI replaced ultrasound endoscopy as an imaging modality. Patients with a positive mesorectal fascia (MRF), but also very distal tumours, could benefit from higher total doses with a longer interval and adding chemotherapy. This facilitated tumour shrinkage and, in theory, in a proportion of patients, also sphincter-sparing surgery. After short-course radiotherapy, surgery was performed almost immediately after the end of radiotherapy, so here, no benefit in downstaging was to be expected.

The German Colorectal Study group performed an important trial, randomising between postoperative chemoradiation (CRT) and preoperative CRT for patients with T3 or T4 or node-positive disease [34]. Similar to results in the Uppsala trial, radiotherapy in a preoperative setting was superior in the local control, as were compliance and toxicity, but it could not show a benefit in terms of OS. The local relapse rate was 6% in the preoperative group vs. 13% in the postoperative group (*p* = 0.006). Preoperative CRT was associated with reduced toxicity, both short- and long-term. In the same period, two large trials, EORTC-22921 and FFCD, confirmed the positive effect of CRT on local control, both comparing conventional long-course radiotherapy with CRT [35,36]. 

Although CRT has been proven to downsize and -stage tumours, even to a complete response, a very clear effect on sphincter-preserving surgery and the negative resection margin has not been proven. In the German trial, no significant difference in overall sphincter-preserving surgery was found after preoperative CRT. However, a subgroup analysis showed that in the preoperative group, 39% of patients (45/116) for whom, at baseline, an APR resection had been deemed necessary could have sphincter-preserving surgery, compared to 19% in the postoperative group (*p* = 0.004). A Polish trial specifically investigated sphincter-preserving surgery and found no significant difference in sphincter-preserving surgery rates between CRT with a waiting interval and short-course radiotherapy (SCRT, with immediate surgery, so no downstaging was expected) for T3 and T4 tumours [37]. This was confirmed by an Australian trial with similar treatment arms [38]. The addition of 5-FU to long-course radiotherapy in the FFCD trial also did not show a difference in sphincter preservation [35] while the EORTC-22921 reported a marginally increased rate of anterior resection from 52.6% to 55.6% (*p* = 0.05) [39]. Important to note, however, is that comparison is difficult with differences in tumour locations and stages between the trials. Additionally, TME surgery was not yet being performed routinely, and the improvement of surgical techniques has played an important role in decreasing the usage of APR procedures over time, thus blurring the net effect of (neo)adjuvant treatment on sphincter preservation rates [2].

The fact that all these trials did report a significant benefit of CRT in terms of downstaging and achieving a pathological complete response (pCR) when compared to their control arm suggests that downsizing does happen, but only in select cases where it is enough to adjust the surgical plan.

Postponing surgery for a six-week interval to allow tumour shrinkage and recovery from side effects did not result in an increased rate of surgical complications in the German trial (36% after preoperative CRT vs. 34% after postoperative CRT (*p* = 0.68)) [34]. Also, no difference was reported in the occurrence of anastomotic leakage, delayed sacral-wound healing, postoperative bleeding, and ileus. In the long term, a significant effect was seen in strictures at the anastomotic site (4% preoperative vs. 12% postoperative, *p* = 0.003). The incidence of small bowel obstruction requiring reoperation was 2% vs. 1%. 

## 5. Timing

The timing of surgery is an important factor in surgical outcomes and a matter of debate. A longer delay could, in theory, lead to more technically demanding procedures as a result of fibrosis, leading to worse TME quality and higher complication rates. On the other hand, delaying surgery gives higher chances of pCR and downsizing the tumour. 

The TIMING trial was set up to determine whether a longer interval and adding chemotherapy during the waiting interval would increase response rates after CRT [40]. In addition to a modest increase in the pCR rate, delaying surgery by up to 20 weeks did not increase the occurrence of surgical complications like ileus or infection. And although the occurrence of pelvic fibrosis was increased in the group with a longer interval, there was no difference in the surgeons’ reported technical difficulty. This has also been described in the GRECCAR-6 trial, where patients were randomised for a 7- or 11-week interval after CRT [41]. The quality of the mesorectum was worse in the 11-week group (complete mesorectum, 78.7% vs. 90%, *p* = 0.016), with more pelvic fibrosis described and a longer mean operative time of 15 min. In contrast to the TIMING trial, the GREGGAR-6 trial did find a significant increase in postoperative morbidity after a longer waiting interval (32% vs. 44.5%, *p* = 0.04), with more medical postoperative complications (primarily urinary complications and vein thrombosis). Additionally, a trend was seen towards more perineal complications with a longer waiting interval after APR, although this was not significant (16.7% vs. 42.9%, *p* = 0.310). 

**Figure 1:**
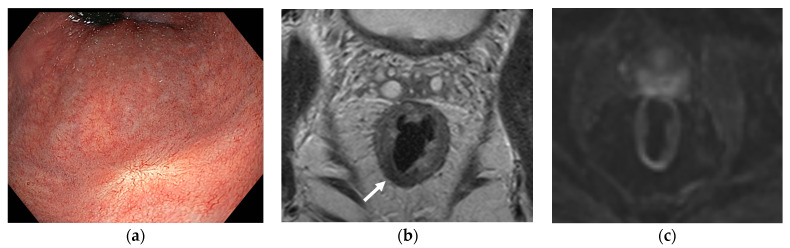
Clinical complete response (cCR) seen via (**a**) endoscopy with a typical white scar, (**b**) magnetic resonance imaging (MRI) with axial T2-weighted imaging with residual fibrosis (white arrow), and (**c**) diffusion-weighted MRI without high signal.

More recently, it became clear that delayed surgery after SCRT was feasible as well. In the third Stockholm non-inferiority trial (Stockholm III), conventional long-course radiotherapy with delay (4–8 weeks), SCRT with delay (4–8 weeks), and SCRT without delay were compared [32]. No oncological differences were found between any of these groups. Even though the trial had a long inclusion period and was underpowered for the primary endpoint, it has had a significant impact on clinical practice. In this trial, fewer postoperative and surgical complications were found after SCRT when delayed surgery was compared to immediate surgery. Since then, clinical practice has gradually shifted towards a delay of at least six weeks after SCRT [3,42]. SCRT with delay had a higher negative resection margin rate of 96.0% compared to 92.3% after immediate surgery, although, between the three groups, this difference was not statistically significant [43]. An added benefit of delaying surgery is potential organ preservation in case a clinical complete response (cCR) is detected at restaging (Figure 1). SCRT with delaying surgery is mostly used in Europe while in the US, the standard of care for stage II and III rectal cancer is CRT or TNT [6,44].

## 6. Total Neoadjuvant Treatment

### 6.1. Oncological Outcomes

Preoperative radiotherapy has had a clear positive effect on local control but has not decreased the number of distant recurrences, and after the introduction of TME surgery, no trial has been able to prove an effect on overall survival. The rationale behind TNT is earlier and superior systemic control with preoperative systemic chemotherapy. Other benefits of TNT include better tumour regression and compliance. 

Trials investigating TNT are of recent date, and although long-term survival results are still lacking, available reports show promising results. Three early trials comparing TNT with CRT are the Polish trial (SCRT + chemotherapy vs. CRT), RAPIDO trial (SCRT + chemotherapy vs. CRT and adjuvant chemotherapy) and PRODIGE trial (chemotherapy+ CRT vs. CRT). In the Polish trial, patients in the TNT arm received three cycles of FOLFOX4 after 5 **×** 5 Gy while in the RAPIDO trial, they received six cycles of CAPOX or nine cycles of FOLFOX4. In the PRODIGE trial, patients first received chemotherapy in the form of six cycles of FOLFIRINOX followed by CRT. The RAPIDO trial found a lower distant metastasis rate in the experimental group but more local recurrences [7,45]. With a double pCR rate in the experimental arm, these results are puzzling, and in-depth analyses are ongoing. The more recent STELLAR trial, with four cycles of chemotherapy, was the only trial that reported a difference in overall survival (86.5% vs. 75.1%, *p* = 0.033) at 3 years, although this should be interpreted with care since no difference in local or distant recurrence was found [46]. At 7 years, the PRODIGE trial has now reported an improvement in OS as well (abstract) [47]. The Polish trial has recently reported no significant difference in OS at 7 years [48]. 

More recent TNT trials have compared the sequence of TNT regimens including CRT, where consolidation chemotherapy (CRT followed by chemotherapy) seems to be the preferred strategy. In the German Colorectal Study group (CAO/ARO/AIO-12), consolidation chemotherapy was better in terms of the pCR rate and compliance whereas no difference was found in DFS, DM, and LR [49]. The same was also investigated in the US in the OPRA trial, which was the first to implement organ preservation in the study protocol [50]. Similar to the German study, no differences were found in oncological outcomes between consolidation and induction chemotherapy, but significantly higher rates of organ preservation were observed in the consolidation arm, with more than half of the patients having preserved their rectums at a median of 3-year follow-up. Since the total dose of radiation and chemotherapy, overall treatment time, and time interval to reassessment were similar in both groups, it is not completely clear why a higher organ preservation rate was seen in the consolidation arm. 

### 6.2. Surgical Outcomes

Compared to CRT, a TNT regimen has an even higher toxicity and a longer overall treatment time, which could, in theory, worsen surgical complications or difficulty but also positively affect downsizing. Only the Polish trial reported a difference in the R0 resection rate of 71% in the experimental arm vs. 77% in the standard arm (*p* = 0.07). This difference was not found in other studies, in which R0 resections in general were significantly higher than in the Polish trial. PRODIGE reported R0 resections of 95% and 94% while the RAPIDO trial reported 90% in both. However, in the latter, more often, a breached mesorectum in the EXP group was reported (according to the surgeon), with a rate of 9% vs. 6% in the STD group (*p* = 0.032). A systematic minireview including trials with TNT vs. CRT confirmed this similarity in resection limits [51]. 

No significant increases in the number of perioperative complications have been reported after TNT. The RAPIDO trial reported no differences in surgical complications within 30 days of surgery, graded according to the Clavien–Dindo classification. Anastomotic leakage was 3% in the EXP arm vs. 2% in the STD arm. The PRODIGE 23 trial reported no difference in the overall percentage of complications, but did report that significantly more grade IV and V complications occurred in the standard-of-care group (0.9% vs. 4.6%, *p* = 0.036) while overall postoperative morbidity showed similar percentages of 29% vs. 31%. The STELLAR trial also reported no differences in the prevalence of grade III+ surgical complications. 

Compared to the earlier radiotherapy trials, the postoperative mortality rates have significantly improved, presumably due to more dedicated colorectal surgery including laparoscopic and robotic procedures, better perioperative care, and optimised prehabilitation. The PRODIGE trial reported postoperative mortality rates within 30 days of 0% after TNT vs. 2.3% after CRT (*p* = 0.061) [8] while in the RAPIDO trial, this was <1% in both arms [52]. 

No differences between TNT and CRT were found in terms of sphincter-preserving surgery or diverting ileostomy rates. The PRODIGE trial also reported no differences in the type of surgery, with an APR rate of 14% in both arms, and no difference in permanent stoma rates. APR rates in the STELLAR trial were 45% and 41% [46]. 

Right now, NCCN guidelines recommend TNT for all patients with more advanced clinical stages as risk factors are identified to individualize patients more likely to benefit from TNT [53].

## 7. Future Perspectives

### 7.1. Non-Operative Management

A major advantage of TNT includes the high percentages of pCR rates, which have led to study designs implementing intentional organ preservation in their protocols [50]. After a cCR, a W&W strategy can avoid an APR and (major) LARS after rectal resection with primary anastomosis. However, it is important to note that radiation-induced toxicity can also severely impact long-term bowel function and patients’ quality of life [29,54]. A large prospective cohort study with patients who followed a W&W approach after radiotherapy showed that around 25% of patients still reported major bowel dysfunction [55]. Quality of life scores became significantly worse after TME surgery was needed. 

Implementing organ preservation in prospective clinical trials was not common until recently. However, pCR rates have been reported with increasing interest since pCR rates differ considerably depending on the type of neoadjuvant treatment given. After SCRT with delayed surgery, the pCR rate in the Stockholm III trial was 10%, and after CRT, rates of 9–14% have been reported [40,43]. The highest rates have been reported after TNT, with pCR rates doubling compared to CRT to around 28% [7,49]. The OPRA trial was the first to implement organ preservation, and in the consolidation arm, TME-free survival was as high as 50% [50]. However, although surgical morbidity will be decreased due to omitting surgery, the short- and long-term toxicity of the treatment seem increased after TNT [52,56]. Ongoing trials are investigating different TNT regimens with organ preservation as a primary endpoint. The German ACO/ARO/AIO-18.1 compares SCRT and CRT, both followed by consolidation chemotherapy and surgery, and a W&W approach in case of a cCR [57]. 

The standard of care for early-stage rectal cancer does not include radiotherapy and patients would therefore never achieve a complete response. Recent trials have started investigating techniques to facilitate organ preservation also in earlier tumours while, at the same time, advanced endoscopy techniques like endoscopic submucosal and intermuscular dissection (ESD and EID) are increasingly used. In particular, for elderly and frail patients not fit for surgery, this planned form of organ preservation could be a good alternative. The OPERA trial included patients with small tumours who, after having CRT, received either external beam radiotherapy or contact X-ray brachytherapy [58]. In particular, tumours smaller than 3 cm in size had an impressive organ preservation rate of 97% when first having X-ray brachytherapy followed by CRT. Results from the STAR-TREC trial are awaited; in this trial, patients with cT1-3b tumours ≤40 mm in size are included into either the standard arm with only TME surgery, 5 × 5 Gy arm, or CRT arm [59]. In case of a good but incomplete response, transanal endoscopic microsurgery (TEM) can be used to excise remaining tumours. The ongoing OPAXX trial investigates the role of contact X-ray brachytherapy and local excision in case of a good clinical response after CRT [60]. 

### 7.2. Patient Selection

Since the landmark trials in the 1990s, perioperative management has evolved, including the improvement of preoperative staging and imaging. Nowadays, MRI is the standard of care, making it easier to distinguish prognostic factors like T-stage, and nodal, MRF and extramural vascular invasion (EMVI) involvement. Consequently, the type of neoadjuvant treatment can be chosen more precisely based on the clinical tumour stage. 

The recent PROSPECT trial selected patients for additional neoadjuvant treatment depending on the degree of tumour regression [61]. Patients who were up for sphincter-sparing surgery were randomised in either the standard of care, CRT, or systemic chemotherapy (FOLFOX) with the addition of CRT only when the tumour had not decreased enough (<20%). Recent results showed that this approach was non-inferior to CRT in terms of DFS and OS. These are interesting results, indicating that isolated systemic therapy also achieves considerable pCR rates (22% in FOLFOX group vs. 24% in CRT group) while patients can be saved from radiotherapy toxicity. However, a longer follow-up is required to evaluate late effects. 

Up to date, there have been no clear biomarkers used in clinical practice to personalise rectal cancer treatment. Circulating tumour DNA (ctDNA) has been shown to have a strong association with residual disease and unfavourable prognosis. Although not yet investigated in a randomised setting, it could therefore be used as guidance in therapy selection. For mismatch repair-deficient (dMMR) rectal cancer, Cercek et al. have recently published promising results, using induction PD-1 blockade as an neoadjuvant treatment [62]. In this phase II trial, 100% of 12 patients had a sustained clinical complete response after at least 6 months of follow-up. Unfortunately, only a very small percentage of rectal cancer patients are MMR-deficient. Ideally, in the future, more distinctive molecular features can be determined to predict response, leading to a reduction in overtreatment. 

## 8. Conclusions

In conclusion, in the last few decades, surgical morbidity and mortality have significantly been reduced due to the implementation of TME surgery. The intensification of neoadjuvant regimens and longer overall treatment times increase pCR rates and organ preservation rates without evident detrimental effects on surgical outcomes. With research more focused on organ preservation also in nonlocally advanced rectal cancer, multidisciplinary discussion should include the patient preference, the treatment toxicity, the tumour height, the predicted functional outcome after rectal anastomosis, and the likelihood of end colostomy. Finally, also, the burden of intensive surveillance in a W&W program deserves proper attention.
